# Optimization of Phenotyping Assays for the Model Monocot *Setaria viridis*

**DOI:** 10.3389/fpls.2017.02172

**Published:** 2017-12-22

**Authors:** Biswa R. Acharya, Swarup Roy Choudhury, Aiden B. Estelle, Anitha Vijayakumar, Chuanmei Zhu, Laryssa Hovis, Sona Pandey

**Affiliations:** Donald Danforth Plant Science Center, St. Louis, MO, United States

**Keywords:** *Setaria viridis*, seedling growth and development, seed germination, phytohormones, abiotic stress, phenotyping

## Abstract

*Setaria viridis* (green foxtail) is an important model plant for the study of C4 photosynthesis in panicoid grasses, and is fast emerging as a system of choice for the study of plant development, domestication, abiotic stress responses and evolution. Basic research findings in Setaria are expected to advance research not only in this species and its close relative *S. italica* (foxtail millet), but also in other panicoid grasses, many of which are important food or bioenergy crops. Here we report on the standardization of multiple growth and development assays for *S. viridis* under controlled conditions, and in response to several phytohormones and abiotic stresses. We optimized these assays at three different stages of the plant’s life: seed germination and post-germination growth using agar plate-based assays, early seedling growth and development using germination pouch-based assays, and adult plant growth and development under environmentally controlled growth chambers and greenhouses. These assays will be useful for the community to perform large scale phenotyping analyses, mutant screens, comparative physiological analysis, and functional characterization of novel genes of *Setaria* or other related agricultural crops. Precise description of various growth conditions, effective treatment conditions and description of the resultant phenotypes will help expand the use of *S. viridis* as an effective model system.

## Introduction

*Setaria viridis* (Setaria, hereafter), a C4 plant, belongs to the grass subfamily Panicoideae which also includes important crops like maize, sugarcane, sorghum and potential biofuel species such as Miscanthus and switchgrass. Setaria possesses all inherent characteristics of an ideal reference plant; the plants are easy to grow and maintain, have small stature (∼30 cm at maturity), rapid life cycle (6–8 weeks), produce large quantities of seed and are diploid with a small genome size of ∼500 Mb (Brutnell et al., 2010). The Setaria genome has been sequenced and *Agrobacterium*-mediated Setaria callus transformation and spike–dip transformation methods have been established (Bennetzen et al., 2012; Martins et al., 2015; Saha and Blumwald, 2016). There is large extant genetic diversity in this species and mapping populations and large-scale mutant populations are being developed (Huang et al., 2014, 2017; Layton and Kellogg, 2014; Schroder et al., 2017). Together with its close relative *S. italica*, with which it is interfertile, *S. viridis* also serves as a good model system to study crop domestication (Li and Brutnell, 2011). Furthermore, extensive genomics and transcriptomics resources are currently available and are being constantly developed for this species (Xu J. et al., 2013; Huang et al., 2016; Singh et al., 2016; Pandey et al., 2017). A number of studies, many already harnessing these resources, have recently been published corroborating its considerable potential as a model system for gene discovery, physiological and developmental analyses, stress responses and nutrient use efficiency (Yue et al., 2014; Li et al., 2016; Mauro-Herrera and Doust, 2016; Pan et al., 2016; Bandyopadhyay et al., 2017; Huang et al., 2017). Given its similarity to and close phylogenetic relationship with a number of important food crops, it is expected that identification of novel gene functions in *S. viridis* that are associated with growth and development, grain size, yield and biomass, resistance to different pathogens and abiotic factors will have huge benefits for translational research geared toward improving food security.

Despite the progress made with resource development for Setaria, and its increasing usage across the world as a model system for the study of panicoid grasses, critical aspects of its growth and development and the factors affecting them are not as well-established as for other model plants such as Arabidopsis. The development of standardized and simple methods to perform efficient genetic and physiological analysis is critical at this stage to expand its use as an effective model species, as well as for comparing results across laboratories. Moreover, development of fast, efficient and standardized seed or seedling-based assays will also be useful for large-scale mutant screen or bulk segregant analysis, for the evaluation of abiotic or biotic stress responses of plants, and to study the effect of different plant hormones or other additives. Toward this goal, we have standardized a set of assays which describe optimum growth conditions for dormancy breakage and seed germination, early seedling growth and development and adult plant growth phenotypes for Setaria.

Seed germination is an important biological process in the plant life cycle which is tightly regulated by the antagonistic phytohormones abscisic acid (ABA) and gibberellic acid (GA_3_). ABA serves as a negative regulator of seed germination whereas GA_3_ is a positive regulator and their dynamic metabolic status determines whether a dormant seed will germinate or not (Finkelstein et al., 2008; Nonogaki, 2014; Gazzarrini and Tsai, 2015; Shu et al., 2015, 2016). In addition to hormonal signals, environmental cues such as light, temperature, and water also impact seed germination (Nonogaki, 2014; Gazzarrini and Tsai, 2015; Shu et al., 2015). Seed germination assays are a quick, easy and efficient way to evaluate seed dormancy, seed quality, effect of different phytohormones, response to various abiotic stresses and other environmental conditions. Many of the key Arabidopsis mutants, especially those related to ABA, GA, and ethylene signaling and metabolism were identified through seed germination screens (Bleecker et al., 1988; Guzman and Ecker, 1990; Saleki et al., 1993; Yang et al., 1995; Leon-Kloosterziel et al., 1996; Ogawa et al., 2003). These screens are usually performed with plate-based assays, which allow for easy, efficient screening of thousands of seeds in a short time. Similarly, post-germination growth and early-seedling growth assays are also key indicators of the plants’ response to a multitude of environmental, abiotic and biotic stress signals and can be efficiently applied to large plant populations. Even though it is expected that different environmental signals, phytohormones or stresses will affect Setaria growth and development, a detailed analysis of those effects, the effective concentration ranges for different additives and the critical stages for observing specific plant phenotypes remain underexplored. Often such information is derived from more established model systems such as Arabidopsis, which may not be optimal in this case.

Several studies have reported on different aspects of seed germination in Setaria, including comparisons between different accessions or species, interspecies comparison of germination efficiencies and the effects of different environmental conditions on seed germination (Manthey and Nalewaja, 1987; Dekker and Hargrove, 2002; Liu et al., 2003, 2017; Mauro-Herrera et al., 2013; Sebastian et al., 2014; Amini et al., 2015a,b; Shu et al., 2015; Saha and Blumwald, 2016; Saha et al., 2016; Hodge and Doust, 2017). Assays have also been developed for germination on synthetic media plates under controlled conditions and genetic basis of seed germination efficiency has been explored (Mauro-Herrera et al., 2013; Qie et al., 2014; Sebastian et al., 2014; Muthamilarasan et al., 2015; Feng et al., 2016; Feldman et al., 2017; Huang et al., 2017; Liu et al., 2017); all with the aim of effectively breaking the seed dormancy, which remains a major problem in Setaria. Freshly harvested Setaria seeds are difficult to germinate on synthetic media and typically require various pretreatments such as long post-harvest storage (1–3 months), a cold shock treatment, liquid smoke or gibberellic acid treatment, etc. (Mauro-Herrera et al., 2013; Sebastian et al., 2014; Saha and Blumwald, 2016; Saha et al., 2016). Even under these conditions the consistent, homogenous germination is relatively difficult to achieve, which may be important for many physiological assays.

The goals of this research were to identify suitable conditions for breaking dormancy of Setaria seeds and to standardize media and growth conditions for efficient plate-based assays. In addition, the effect of each of the major phytohormones was analyzed on germination and/or on early seedling growth. Adult plants were also compared for growth in different conditions especially in the context of their photoperiod requirement, nutrient requirement and water stress response. The results presented in the following sections describe a set of optimum conditions which will be useful for the community at large for multiple phenotypic analyses and will help to improve the use of Setaria as a model system for important food, feed and fuel crops.

## Materials and Methods

### Seed Selection, Liquid Smoke Treatment, and Seed Sterilization

*Setaria viridis* accession A10.1 is used for all assays described in this report, with accession ME034V also being tested in controlled growth chambers and greenhouse conditions for comparison. After seed collection, the papery glume and lower lemma were removed and black or dark brown seeds were separated from pale or greenish seeds, which are likely a mix of both mature and immature seeds. Darker seeds consistently showed better germination and have been used for the assays described in this report. Two different sterilization protocols were tested. In one, seed dormancy was broken by treating seeds with Hickory liquid smoke obtained from a local grocery store at or 5% for 24 h (Sebastian et al., 2014) or 50% for 2 h (Brutnell Lab, Personal Communication) followed by five washes with autoclaved MQ water to remove liquid smoke. Seeds were then surface-sterilized with 20% bleach (Clorox) and 0.1% Tween-20 (Sigma-Aldrich) for 20 min with continuous shaking. Alternatively, seeds were treated with 20% bleach and 0.1% Tween-20 for 1 h with continuous shaking without liquid smoke treatment. The sterilized seeds were washed eight times with autoclaved MQ water and plated on 0.5X MS media (Caisson Labs, Smithfield, UT, United States) plates or inserted in seed germination pouches (PhytoAB, Redwood City, CA, United States; Catalogue No. CYG-38LB). Seeds sown on plates were stratified at 4°C in the dark for 2 days followed by transfer to controlled growth chamber with 12 h day/night (31°C/22°C) regime, with light intensity 450 μmol/m^2^/sec and relative humidity 50–60%. Seeds sown on germination pouches or soil were transferred to growth chambers or greenhouse without any need of stratification. For growth in soil, seeds without sterilization were sown in 10 cm pots containing Metromix 360 (Hummert International, Earth City, MO, United States) and transferred to controlled growth chambers with 12 h day/night (31°C/22°C) regime, with light intensity 450 μmol/m^2^/sec and relative humidity 50–60% or in greenhouses (28°C/22°C (day/night) temperature, 30–50% humidity, ∼300–450 μmol/m^2^/sec light intensity depending on the weather and time of the day). In greenhouses, supplemental lighting was applied when the outside light level is <600 watts/sq. meter/sec. From May through September, supplemental lighting was available between 06:00 and 10:00 am. October through April it was available from 06:00 am to 8:00 pm. The lights are a mix of metal halide and high pressure sodium, and provide a minimum of 300 μmol/m^2^/sec. When the outside light is >1000 W/sq. meter/sec., the shade cloth was pulled. Relative humidity was maintained at a minimum of 30%.

### Seed Germination Assays

Seed germination assays were performed on 0.5X MS media plates. Germination was defined as radicle emergence which was recorded from day 1 to day 4 post-stratification (Hodge and Doust, 2017). For plate-based assays we evaluated the germination of 3 months old versus freshly harvested seeds, the effect of GA_3_ (Caisson Labs) on the germination of freshly harvested seeds, the effect of agar (0.8%) or phytagel (0.4%) as gelling agents (Caisson Labs), the effect of liquid smoke treatment, and the effect of different sucrose concentrations (0, 0.5, 1.0, and 1.5% sucrose) in the media. To assess the effect of different stresses during germination, seeds were plated directly on media containing different concentrations of ABA (Caisson Labs), NaCl, or glucose.

### Post-germination and Early Seedling Growth Assays

Effect of different phytohormones was assessed during post-germination, early seedling growth in both plate-based assays and in germination pouches. For plate-based assays, seeds were placed on media containing different concentrations of brassinosteroids (brassinolide, BL, Sigma-Aldrich, St. Louis, MO, United States), ethylene (ACC, ethylene precursor, Sigma), jasmonic acid (JA, Sigma) and auxin (IAA, indole-3- acetic acid, Caisson Labs) followed by stratification for two days at 4°C in the darkness. Plates were transferred to the growth chambers and incubated in light for 6 h to help germination. Plates were then covered with aluminum foil (except for auxin treatment) and seedlings were allowed to grow for 4–5 days. Seed germination percentage, root lengths, and coleoptile lengths were quantified. To evaluate the effect of ABA, NaCl, and glucose on post-germination growth and root length, seeds were first germinated on control media and then transferred to media plates supplemented with different concentrations of desired additives. Seedlings were grown in the 12 h dark/12 h light cycle and coleoptile lengths, root lengths, and secondary root numbers were measured from day 1 to day 7 post-transfer for each of the treatments.

For assays using germination pouches, seeds were germinated under control conditions by placing them on pouches pre-wet with autoclaved water. The water in germination pouches was replaced with desired media or additives 48 h post-imbibition. Seedlings were allowed to grow for 2 weeks. Root lengths were recorded every day.

### Adult Plant Growth, Phenotyping, and Stress Treatment

To compare the growth of plants between greenhouses and growth chambers, seedlings were grown under similar conditions (except day length, which was maintained in the growth chambers at 12 h day/12 h night). To analyze the effect of nitrogen and water deficit, plants were grown under four different conditions; well-watered with sufficient nitrogen (W), well-watered, without nitrogen (W-N), low water, sufficient nitrogen (L), and low water without nitrogen (L-N). The amount of water need to be supplemented to the plant survival was determined prior to the experiment. Both W and L were provided with the nutrient medium supplemented with 15 mM nitrogen (added as KNO_3_) whereas in the treatments without nitrogen (W-N and L-N) no exogenous nitrogen was added in the nutrient media. The L and L-N plants were provided with 50% the amount of water as compared to the W plants. All four treatments were conducted at the same time in the greenhouse with 50% humidity and 31°C/22°C (day/night) temperature. Four plants and three replicates were used for each treatment. Multiple growth parameters, including plant height, leaf number, day to heading, panicle number, and seed yield were recorded periodically starting from one-week post-germination till the plants matured and dried.

### Statistical Analysis

All experiments were repeated at least three times independently and data were averaged. Means, standard deviation (seed germination assays) and standard errors (for root length and number, coleoptile length, leaf number, plant height, panicle number, and size and seed weight) for measurements were calculated. Statistical significance of results was calculated using Student’s *t*-test with a *P*-value threshold of less than 0.05.

## Results and Discussion

### Standardization of Setaria Seed Germination Conditions

**FIGURE 1:**
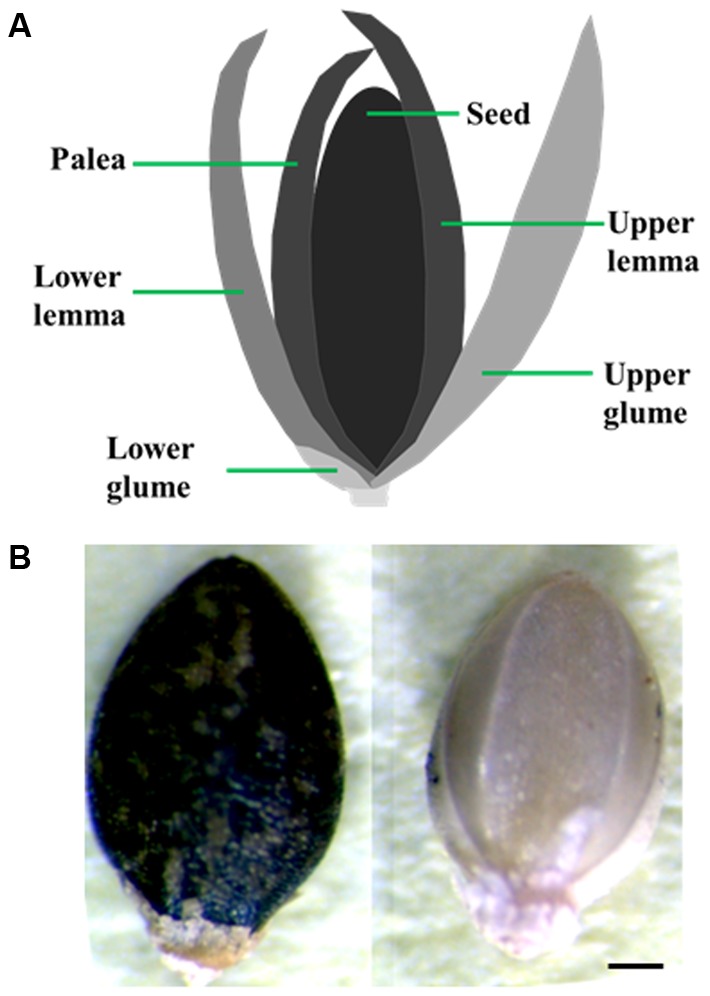
Seeds used for germination and growth assays. **(A)** Diagrammatic representation of a Setaria floret. Upper anthecium which consists of hard upper lemma and palea attached to the caryopsis (fruit) was used in germination and growth assays. **(B)** Dark and pale seeds isolated form the same panicle. Dark seeds were used for all experiments. Scale bar is 250 μm.

**FIGURE 2:**
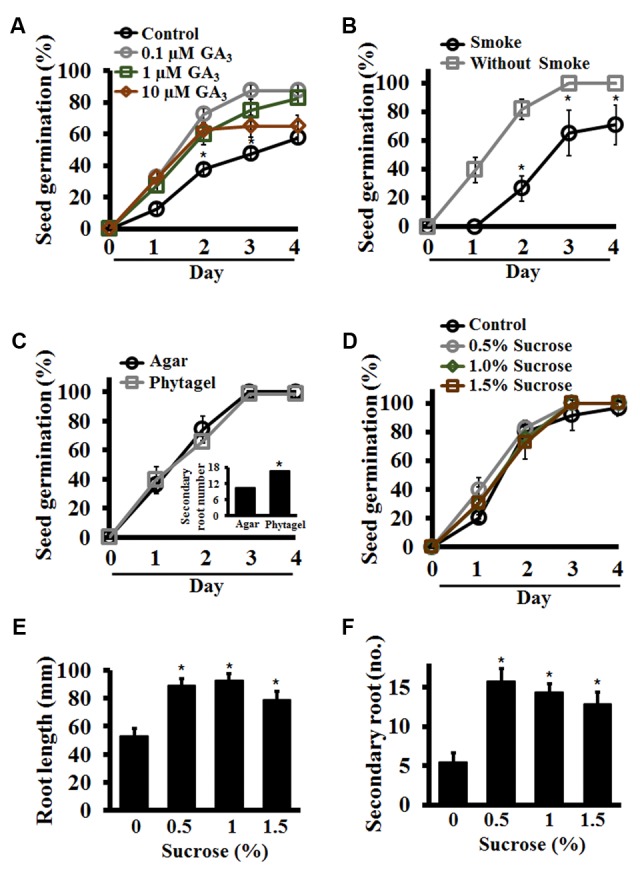
Standardization of seed germination conditions for Setaria using synthetic media in plate-based assays. **(A)** Germination percentage of freshly harvested Setaria seeds was assessed in the presence of varying concentrations of exogenously added gibberellic acid (GA_3_). **(B)** Seeds were treated with or without liquid smoke before sterilization and germination percentage was quantified from day 1 to day 4. **(C)** Percentage of seed germination of Setaria was counted from day 1 to day 4 on Phytagel- or Agar-based 0.5X MS media. The number of secondary roots at day 7 was also quantified (inset panel). **(D)** Effect of varying concentrations of sucrose on seed germination of Setaria was quantified for up to 4 days post-stratification. **(E)** The primary root length of Setaria seedlings were measured at 7 day post-stratification in the presence of varying concentrations of sucrose. **(F)** Secondary root number of Setaria seedlings was quantified at 7 day post-stratification. In all experiments, seeds sown on 0.5X MS media plates were stratified at 4 °C in the dark for 2 days followed by transfer to the growth chamber with light-12 h (31°C)/dark-12 h (22°C) cycle. Plates were placed vertically to measure the root length and secondary root number. Minimum 3 months old, dark-colored seeds were used in the experiments **(C–F)**. Data were averaged from three biological replicates and at least 60–70 seeds were used in each experiment. For **(A–D)**, error bars represent standard deviation (±SD). For **(E,F)** Error bars represent standard error (±SE). Asterisks represent *P*-values less than 0.05 as calculated using Student’s *t*-test.

Optimization of conditions that result in uniform, 100% seed germination under a controlled environment is crucial for the success of many phenotypic assays. Achieving uniform germination in *S. viridis* has been a challenge as it displays huge discrepancies depending on the age of the seeds, dormancy breaking requirements and appropriate media and environmental conditions (Nonogaki, 2014; Sebastian et al., 2014; Saha et al., 2016; Hodge and Doust, 2017). To address this, we performed a set of assays to identify conditions that result in consistent, homogenous germination of seeds. All through this manuscript we have used the upper anthecium, i.e., entire upper floret that contains the caryopsis (fruit) with upper lemma and palea attached to it (**Figure 1A**) and referred to it as seed for the sake of simplicity. Our results show that seed quality and age have a huge effect on germination potential of Setaria seeds. First, seeds which were darker in color with glume and lower lemma removed from them show significantly improved, uniform germination compared to pale or green seeds harvested from the same panicle (**Figure 1B**). We have used dark seeds for all assays described in this report. Secondly, the age of the seeds also has a significant effect as has been previously reported (Sebastian et al., 2014; Hodge and Doust, 2017). In general, seeds stored in the dark at 4°C for at least 3 months after harvesting germinate at 100% efficiency, compared to freshly harvested seeds which show germination efficiency anywhere from 20 to 60%. Treatment of freshly harvested seeds (1-week-old) with exogenous GA_3_ did significantly improve germination. By 4-day post-stratification up to 90% seeds germinated in the presence of 100 nM or 1 μM GA_3_ compared to *ca.* 50% seeds in the absence of exogenous GA_3_ (**Figure 2A**). This concentration of GA_3_ is significantly lower that what has been reported previously (Sebastian et al., 2014). We did not observe any difference in germination efficiency between seeds aged 3 months to 1 year post-harvest. Moreover, liquid smoke treatment on freshly harvested Setaria seeds has been reported to promote germination efficiency (Sebastian et al., 2014). In our experiments, treatment with 5% liquid smoke for 24 h resulted in higher rates of contamination whereas a 50% liquid smoke for 2 h had a negative effect on the germination of 3- to 12-months post-harvest seeds (**Figure 2B**). At 2 days post-stratification, control seeds (without liquid smoke treatment) showed more than 80% seed germination compared to the liquid smoke treatment where less than 25% seeds germinated. By 3 days, all non-treated seeds had germinated, whereas seeds treated with liquid smoke showed 60 and 65% germination on by 3 and 4 days, respectively (**Figure 2B**). We consistently achieved 100% seed germination using 3-month-old, dark seeds with no smoke treatment, which were sterilized with 20% bleach and 0.1% Tween-20 for 1 h with continuous shaking, followed by extensive washing with autoclaved distilled water. Shorter sterilization times (15 or 30 min) resulted in occasional contamination on synthetic media plates or in germination pouches. These conditions were used for all assays described in this manuscript, although additional sterilization protocols have also been reported and may be useful according to the individual requirement (Saha and Blumwald, 2016; Saha et al., 2016).

For plate based assays we further tested seed germination and growth on different concentrations of phytagel- (0.3–1.5%) versus agar- (0.4–1.5%) based MS media, as both these gelling agents have been used previously (Boyes et al., 2001; Xu W. et al., 2013). We found 0.4% phytagel and 0.8% agar based media to be most effective in Setaria seed germination (data not shown). Parallel assays were then performed using both gelling agents to assess seed germination, root length, and secondary root number per seedling. No difference was observed in seed germination efficiency (**Figure 2C**) or root length (not shown) of the seedlings. However, seedlings that were grown on Phytagel-based media showed ∼60% more secondary roots than seedlings that were grown on Agar-based media (**Figure 2C**, inset), indicating that the Phytagel-based media may promote a more robust root system architecture (RSA) in Setaria. We used Phytagel-based media for subsequent assays, although the choice of gelling agent can be varied based on the specific experimental requirements.

We next assessed the amount of sucrose in the germination media as the presence, absence or variation of sucrose content is known to affect germination rate and seedling growth of plants such as Arabidopsis (Finkelstein and Lynch, 2000; Dekkers et al., 2004, 2008). Media without sucrose or supplemented with 0.5, 1.0, and 1.5% sucrose were used to assess optimal sucrose content needed for seed germination and seedling growth. On day 1, different sucrose concentrations had some effect with less than 20% germination on media without sucrose versus ∼40% germination in media with sucrose. However, later on during germination, no significant differences were observed in germination frequency by the presence of sucrose in the media and by day 4 all seeds on all media had germinated (**Figure 2D**). This suggests that for seed germination *per se*, sucrose is not a requirement. However, post-germination growth was significantly affected by the presence of sucrose. Seedlings grown on MS media without sucrose showed smaller primary roots (∼55 mm) whereas the root lengths were ∼90 mm for media containing 0.5 and 1% sucrose and ∼80 mm for media with 1.5% sucrose (**Figure 2E**). The difference in secondary root numbers was also striking. Seedlings grown on media with no additional sucrose developed few secondary roots (5–6 secondary roots/primary root), whereas those growing on media with sucrose developed up to three times more roots (**Figure 2F**). These observations suggest that 0.5–1.0% sucrose containing MS-Phytagel media should be preferred for Setaria seedling growth in plate-based assays. We used 0.5% sucrose in the 0.5X MS, Phytagel media for all the assays described in this report. Identical sucrose concentration has been used in previously reported studies as well (Sebastian et al., 2014).

### Effect of Different Abiotic Stresses on Setaria Seed Germination

**FIGURE 3:**
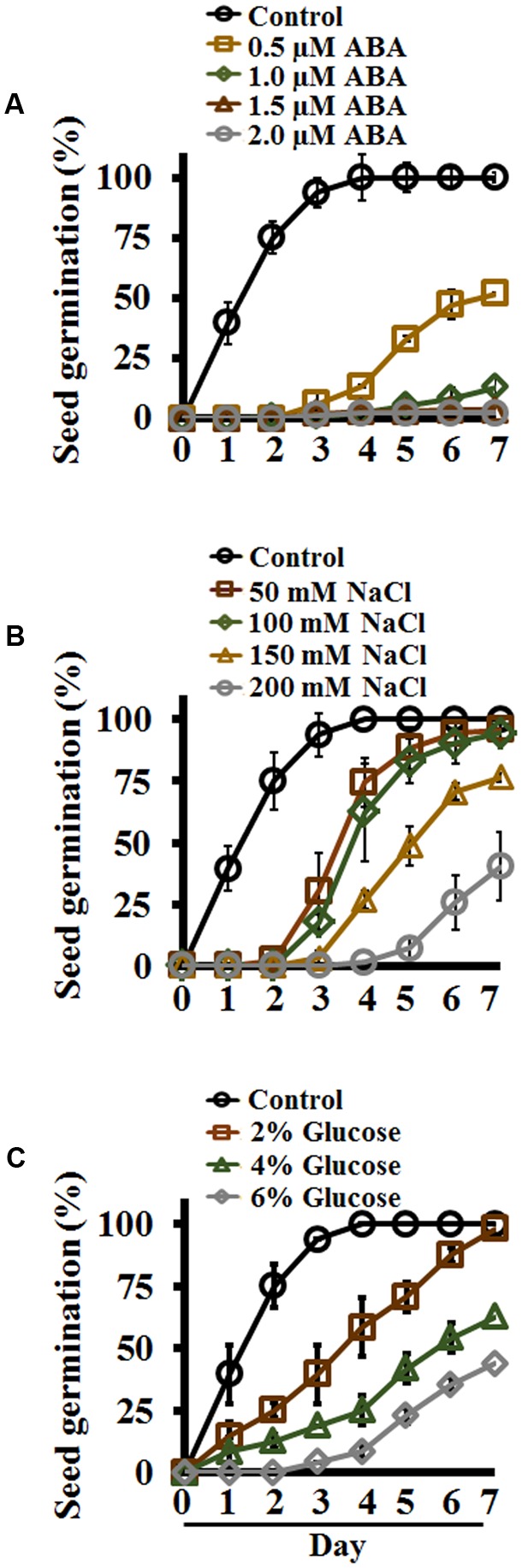
Effect of exogenous abscisic acid (ABA), NaCl and glucose on Setaria seed germination in plate-based assays. Sterilized Setaria seeds were plated on varying concentrations of **(A)** ABA, **(B)** NaCl, and **(C)** glucose. Seed germination (*n* = 60 per treatment) was counted for up to 7 days and compared with germination on control media. Minimum 3 months old, dark-colored seeds were used. In all experiments, seeds were stratified at 4°C in the dark for 2 days followed by transfer to the growth chamber with light-12 h (31°C)/dark-12 h (22°C) cycle. Three biological replicates of each treatment were used for data analysis. Error bars represent standard deviation (±SD).

Several exogenous factors affect seed germination, and germination in the presence of different hormones or stressors has been widely used for screening of mutant seeds, identification of abiotic stress sensitivity of plants, or to study the effect of plant hormones such as ABA, which is well-known to inhibit seed germination (Leon-Kloosterziel et al., 1996; Garciarrubio et al., 1997; Finkelstein et al., 2002, 2008; Brocard-Gifford et al., 2003). Similarly, the effect of different abiotic stresses have been studied on different aspects of Setaria growth and development (Dekker and Hargrove, 2002; Doust and Kellogg, 2006; Yue et al., 2014; Amini et al., 2015b; Muthamilarasan et al., 2015; Feng et al., 2016; Li et al., 2016; Pan et al., 2016; Saha et al., 2016; Singh et al., 2016; Feldman et al., 2017; Pandey et al., 2017). We evaluated the effect of different ABA concentrations ranging from 0.5 to 10 μM on Setaria seed germination. Media plates (0.5X MS media, 0.4% phytagel, 0.5% sucrose) were supplemented with different concentrations of ABA; an equimolar concentration of EtOH (an ABA solvent) was used as a control. Germination was recorded every 24 h post-stratification for 7 days and expressed as percentage of total seeds. Setaria seeds exhibited extreme sensitivity to ABA. More than 75% of seeds had germinated by 2 days on control media, whereas no germination was seen in the presence of ABA even at the lowest (0.5 μM) concentration. By day 3, 90% seed germination was observed on control media, and less than 10% seeds germinated on 0.5 μM ABA with almost no germination at higher ABA concentrations. By day 7, all seeds had germinated on the control media plates; seed germination was ∼40% and ∼10% on media containing 0.5 and 1.0 μM ABA, respectively (**Figure 3A**). One or two seeds occasionally germinated on 1.5 or 2 μM ABA, whereas no germination was ever seen at higher concentrations (5 μM ABA or above). These data show extremely high sensitivity of Setaria seeds for ABA during germination when compared with other model plants such as Arabidopsis, which shows ∼40–50% germination at 1 μM ABA by 48 h (Pandey et al., 2006).

Salinity stress negatively affects seed germination. NaCl is commonly used as an agent to study the effect of salt stress on seed germination, growth and other physiological processes (Saleki et al., 1993; Yue et al., 2014; Duan et al., 2015; Muthamilarasan et al., 2015; Liu et al., 2017; Pandey et al., 2017). To assess the effect of salinity on Setaria seed germination, seeds were plated on media containing 50, 100, 150, and 200 mM NaCl and compared with control media with no added NaCl. As in the ABA experiment, by day 2 more than 75% of the seeds had germinated on control media plates, whereas no germination was seen in the presence of even the lowest NaCl concentrations (**Figure 3B**). Seeds plated on 50 and 100 mM NaCl showed considerably delayed germination (∼20% by day 3, when almost all control seeds had germinated); however, all these seeds eventually germinated by day 7. Seed germination was even more delayed with higher NaCl concentrations, and by day 7 about 75 and 40% seeds germinated on media plates with 150 and 200 mM NaCl, respectively (**Figure 3B**). These data suggest that at lower salt concentrations, the seeds eventually overcome the effects of stress, and are able to germinate although slower than the germination on control plates. However, at more than 100 mM salt concentration, the stress is too severe for the seeds to overcome, and full germination potential was never achieved.

Sugars such as glucose play important regulatory roles during seed germination. High levels of glucose inhibit seed germination and seedling development in Arabidopsis (Dekkers et al., 2004). We tested the effect of different glucose concentrations on Setaria seed germination. Seeds were germinated on different concentrations of glucose (2, 4, and 6% glucose in MS media; no additional sucrose added). Control media had no glucose or sucrose added to it. A clear, concentration-dependent effect of glucose in delaying seed germination was obvious. By day 4, when all control seeds had germinated, only ∼50% of the seeds had germinated on media containing 2% glucose, and significantly fewer on higher glucose concentrations (**Figure 3C**). By day 7 both control and 2% glucose-containing media showed 100% seed germination. Only 50 and 30% seeds germinated by 7 day on media containing 4 and 6% glucose, respectively. The seeds that did germinate on 4% or higher glucose concentrations exhibited high anthocyanin accumulation, showing the effects of stress.

### Effect of Different Hormones and Abiotic Stresses on Post-germination, Early Seedling Growth of Setaria Using Plate-Based Assays

Post-germination, early seedling growth of plants is extremely sensitive to multiple hormones, nutrients and additives. Seedling-stage assays are useful to evaluate the differences in root length, number of secondary roots, coleoptile or shoot length, and can be performed at a relatively large scale using simple plate-based assays. We assessed the effects of different plant hormones and abiotic stresses on early seedling growth of Setaria to determine their effective concentration ranges and the phenotypic differences that arise due to the treatments.

**FIGURE 4:**
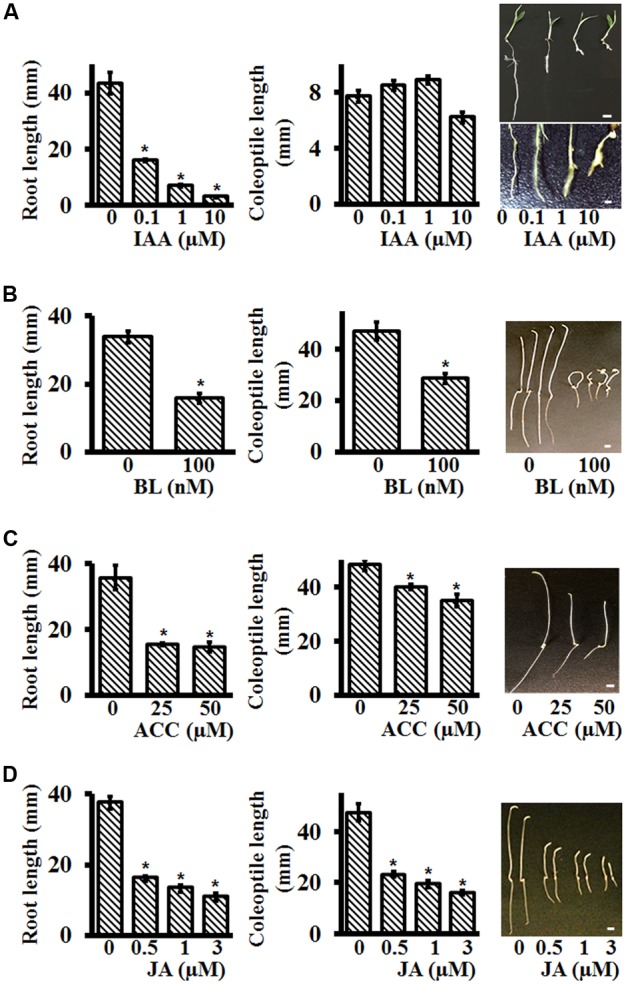
Effect of different plant hormones on post-germination growth of Setaria seedlings using plate-based assays. Sterilized Setaria seeds were plated on varying concentrations of **(A)** indole-3 acetic acid (IAA), **(B)** brassinolide (BL), **(C)** 1-aminocyclopropane-1 carboxylic acid (ACC), and **(D)** methyl jasmonate (JA). The plates were placed vertically. Root and coleoptile lengths were measured after 5 days of growth. For IAA treatment, seedlings were grown in light-12 h (31°C)/dark-12 h (22°C) cycle. The upper and lower panels in the picture show the entire seedlings and terminal root phenotypes (20 X magnification), respectively. For BL, ACC, and JA treatments the seedlings were grown in the dark. The pictures on the right show the phenotypes of 5 day old seedlings in the presence of specific hormone treatments. At least 3 months old, dark-colored seeds were used in the experiment. Data were averaged from three biological replicates. At least 20–30 seeds were used in each experiment. Error bars represent standard error (±SE). For comparative analysis, appropriate controls were included for each treatment. Asterisks represent *P*-values less than 0.05 as calculated using Student’s *t*-test. Scale bars are 5 mm for all figures except for the lower panel of IAA treated roots where it is 1 mm.

Auxins are well-known to affect root growth and development in a variety of plants. In general, auxins inhibit primary root length and promote secondary root formation (Overvoorde et al., 2010). To test the effect of auxins on Setaria roots, seeds were germinated on media plates containing different IAA (Indole-3-acetic acid) concentrations. No effect of IAA was seen on Setaria seed germination. IAA at the concentration range 0.1–10 μM inhibited root growth. Four days post-imbibition, primary root length of seedlings on control, 0.1 and 1 μM IAA was *ca* 43, 16, and 7 mm, respectively (**Figure 4A**). At higher concentrations (10 μM), the roots stopped growing, and were deformed and frequently exhibited bulging (**Figure 4A**). At these concentrations, we did not observe any major differences in secondary root formation between control and IAA treated plants, but at 0.1 μM IAA, the roots were covered with root hairs. The emerging coleoptiles were not significantly different in length at the different IAA concentrations tested (**Figure 4A**).

Brassinosteroids also affect early seedling growth by promoting hypocotyl elongation and root inhibition in plants such as Arabidopsis (Clouse et al., 1996; Clouse, 2001). Although the effects of brassinosteroids are well-documented on later growth stages of monocots such as on lamina joint formation, branching and grain filling, their effects on early seedling growth are not as well-described (Cao and Chen, 1995; Wu et al., 2008; Zhang et al., 2014). Previous studies have reported that in the presence of BL, coleoptile and root growth were inhibited in maize whereas coleoptile growth was promoted (with twisted phenotype) and root growth was inhibited in rice (Yamamuro et al., 2000; Wang et al., 2006; Kir et al., 2015). To test the effect of brassinosteroid on Setaria seedling growth, we grew seeds on different Brassinolide (BL, active brassinosteroid) concentrations, in darkness, and measured primary root and coleoptile lengths. BL at 100 nM caused the most obvious phenotypic differences in the seedlings. After 4 days of post-imbibition growth, 100 nM BL resulted in more than 60% reduction in root length compared to control seedlings (**Figure 4B**). The coleoptiles were also shorter and exhibited the classic twisted, curved growth, as is reported for Arabidopsis seedlings. Not only were seedlings grown on BL stunted but occasionally they also showed tendril like structures on the coleoptiles, a phenotype not reported previously.

The gaseous plant hormone ethylene has a major effect on seedling growth, especially in darkness. Arabidopsis seedlings exhibit a classic triple response in the presence of ethylene or its precursor ACC (1-Aminocyclopropane-1-carboxylic acid), with shorter roots, shorter hypocotyl and apical hook formation (Bleecker et al., 1988; Guzman and Ecker, 1990; Yang et al., 2015). Reduced growth of coleoptiles and roots in the presence of ethylene is also reported for maize, wheat, sorghum and *Brachypodium*, although in rice ethylene is reported to promote coleoptile growth while inhibiting root growth (Satler and Kende, 1985; Ma et al., 2014; Yang et al., 2015). Setaria seedlings exhibited clear differences in root length and coleoptile length in the presence of different concentrations of ACC. ACC concentrations of 25–50 mM inhibited root lengths and coleoptile lengths by more than 50% (**Figure 4C**). However, an apical hook formation, which is typical of Arabidopsis (and other dicot) seedlings was not seen in Setaria. This is likely due to the evolutionary difference between monocots and dicots in how they protect their shoot apical meristem (SAM). As the seeds which are buried under soil germinate during skotomorphogenic developmental program, dicot seedlings protect the SAM by forming an apical hook while monocot seedlings develop a coleoptile for the protection of the SAM (Abbas et al., 2013).

Although jasmonic acid (JA) is usually associated with the defense response of plants, it has a major effect on plant growth and development both directly or by interacting with other plant hormones (Creelman and Mullet, 1995; Turner et al., 2002; Gray, 2004; Wasternack, 2007; Gazzarrini and Tsai, 2015; Shyu and Brutnell, 2015). We evaluated the effect of different concentrations of JA on skotomorphogenic growth of Setaria seedlings. Seeds were plated on control media or on media containing 0.5, 1.0, and 3.0 μM MeJA (methyl jasmonate). Coleoptile and root lengths were measured at 4 days post-imbibition. In comparison to seedlings grown on control media, root lengths were reduced by ∼60, 65, and 70%, and coleoptile lengths were reduced by ∼50, 60, and 65% in seedlings grown in media containing 0.5, 1.0, and 3.0 μM MeJA, respectively (**Figure 4D**). The negative regulatory role of JA during skotomorphogenic growth has been reported previously for the Arabidopsis and maize seedlings (Wasternack, 2007; Shyu and Brutnell, 2015).

Abscisic acid, NaCl and glucose, in addition to exerting a major effect on seed germination, also affect later stages of seedling development (Finkelstein et al., 2002; Dekkers et al., 2008). However, these effects are usually confounded by assay conditions where the seeds are allowed to germinate on media containing these stressors followed by extended growth on the same media. To clearly differentiate the effect of these additives on early seedling growth and development of Setaria, independent of their effects on germination, seeds were first plated on the control media plates and grown for 48 h. By this time all seeds had germinated, as examined by the radicle protrusion from the seeds. The germinated seeds were then transferred to new control media plates or media plates supplemented with different additives.

**FIGURE 5:**
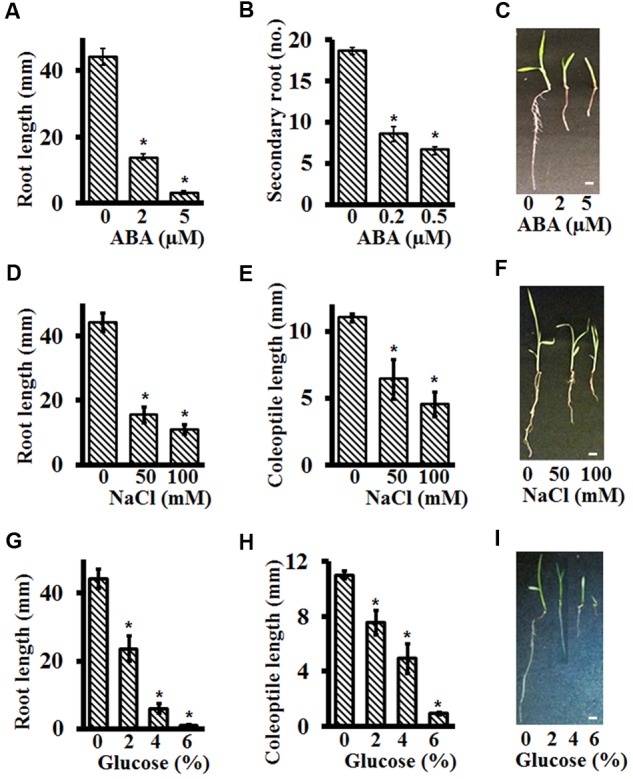
Effect of different stresses on post-germination growth of Setaria using plate-based assays. Sterilized Setaria seeds sown on 0.5X MS media plates were stratified at 4°C in the darkness for 2 days followed by transfer to the growth chamber with light-12 h (31°C)/dark-12 h (22°C) cycle for another 2 days. The effect of ABA on post-germination growth was studied by measuring the primary root length at day 3 **(A)** and secondary root number at day 5 **(B)** after transfer to the media containing varying concentrations of ABA. The picture in **(C)** shows representative seedlings after growth in the presence of ABA. The effect of NaCl **(D,E)** and glucose **(G,H)** was analyzed by quantifying the primary root length and coleoptile length after transfer to the media containing varying concentrations of NaCl and glucose. Figures **(F,I)** show representative images of Setaria seedlings after growth in the presence of NaCl or glucose, respectively. For each experiment, 3 months old, dark-colored seeds were used. Three biological replicates of each treatment were performed and at least 20–30 seeds were used in each experiment. Appropriate controls were used for each treatment. Error bars represent standard errors (±SE). Asterisks represent *P*-values less than 0.05 as calculated using Student’s *t-*test. Scale bars are 5 mm.

Abscisic acid is known to inhibit primary root length and secondary root formation (Harris, 2015). As was seen for seed germination assays, Setaria seedlings were extremely sensitive to ABA for post-germination growth as well. More than 60% reduction was seen in the primary root length at as low as 2 μM ABA, with almost complete growth arrest at 5 μM ABA (**Figure 5A**). This concentration is almost an order of magnitude lower than what is reported for Arabidopsis root growth inhibition assays (Pandey et al., 2006). Secondary root inhibition was also highly sensitive to exogenous ABA and no secondary roots were formed at 1 μM ABA. At concentrations as low as 0.2 μM ABA, more than 50% reduction in secondary root formation was observed (**Figure 5B**). **Figure 5C** shows representative seedlings growing in the presence of ABA.

We also determined the effect of NaCl and glucose on coleoptile and root length inhibition of Setaria seedlings. At 50 mM NaCl, more than 50% reduction in root length (**Figures 5C,E**) and ∼25% reduction in coleoptile length (**Figures 5D,E**) was observed, with higher concentrations (100 mM) leading to stronger effects. For growth in the presence of glucose, as low as 2% glucose had a significant effect on root (>50% reduction) and coleoptile (>25% reduction) lengths (**Figures 5G–I**). It should be noted that at this concentration of glucose, germination *per se*, though delayed, is not affected (**Figure 3C**). Stronger effects were seen at 4% glucose, and seedling growth was almost completely arrested at 6% glucose which could be due to the sugar-induced arrest of seedling development by meristem quiescence (Lastdrager et al., 2014).

### Effect of Different Hormones and Abiotic Stresses on Root Growth of Setaria Using Germination Pouch Assays

**FIGURE 6:**
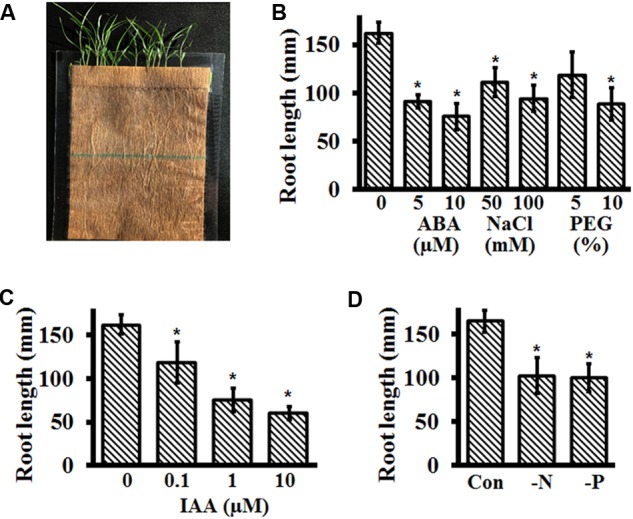
Growth of Setaria in germination pouches and effect of different stresses on seedling growth. **(A)** Representative image of 2-week-old Setaria plants growing in germination pouch. At least 3 months old, dark-colored Setaria seeds were grown under control conditions by placing them on pre-wet pouches. **(B)** For different abiotic stress treatments, seeds were germinated on pre-wet germination pouches for 2 days. The media in the germination pouch was replaced with media supplemented with ABA (5 and 10 μM), NaCl (50 and 100 mM) and PEG (5% and 10%, equivalent to –0.05 and –0.15 MPa osmotic stress). **(C)** The media in germination pouches was replaced with different concentrations of IAA and **(D)** with either minus nitrogen or minus phosphate MS media. In all experiments, the primary root lengths were quantified after 2 weeks of growth. Appropriate controls were used in each experiment **(B–D)**. Three biological replicates of each treatment were performed and at least 20 seeds were used for each experiment. Error bars represent standard errors (±SE). Asterisks represent *P*-values less than 0.05 as calculated using Student’s *t*-test.

Analysis of early seedling growth and development of Setaria on media plates is restricted by time. Even when using 150 mm plates, seedlings could be grown at the most for 1 week. Furthermore, shoot development is not optimal when in the constant contact with media. To address these concerns, we standardized seedling growth assays using seed germination pouches (**Figure 6A**) which resulted in prolific RSA as well as effective growth of shoots exposed to air. We used the medium size pouches (18 cm × 12.5 cm) for Setaria seedling growth and planted 20–22 seeds per pouch. The ability to stack hundreds of pouches in a small container also allowed growing large plant populations in limited space. Additional advantages of growing plants in germination pouches included the ability to change treatment condition without disturbing seedling growth, the ability to monitor and measure root growth at desired time points, and the ability to grow plants without requiring a specialized growth chamber. We successfully grew plants without any special requirements on lab benches (with additional lighting from fluorescent bulbs, ∼150 μmol/m^2^/sec; 16 h/8 h day/night regime) for up to 2 weeks.

We tested the effect of different stresses and hormones on plants growing in germination pouches by first germinating the seeds in 0.5X MS media without sucrose (addition of sucrose results in contamination), followed by replacement with desired media 48 h post-imbibition. Root lengths were recorded every 24 h for 2 weeks. Additionally, because drought and salinity are some of the most critical factors affecting young seedling growth and plants typically respond to these stresses by changing their RSA, we also used the germination pouch assays to assess the effects of water stress on plant growth and development. We present the final root length data for the following experiments, but significant changes in overall RSA and young shoots were obvious by visual inspection and can be quantified as per the specific experimental need.

As expected, treatment with different concentrations of ABA and NaCl significantly inhibited root growth and development (**Figure 6B**). Overall root length of seedlings treated with 5 and 10 μM ABA were ∼55 and 45%, respectively, that of untreated seedlings. This suggests that 5 μM ABA is an optimum concentration to analyze the effect of ABA on Setaria root growth. In the presence of 50 and 100 mM NaCl, the root lengths were inhibited by 25 and 45%, respectively, compared with the untreated seedlings. The effect of glucose cannot be tested in germination pouches, as it results in a high level of contamination (due to growth in non-sterile conditions). However, we did test the effect of polyethylene glycol (PEG8000) as an osmotic stress in this system. Addition of PEG that resulted in an osmotic stress equivalent of -0.05 and -0.15 MPa led to 32 and 43% decrease in root length of Setaria seedlings (**Figure 6B**). Similarly, root growth was also significantly affected by different auxin concentrations (**Figure 6C**), with 1 μM IAA being the optimum.

Besides drought and salinity stresses, nutrient deficiency is a common problem in crop productivity and among the nutrients, nitrogen (N) and phosphorus (P) deficiency are major abiotic stresses that limit plant growth and productivity (Zhao et al., 2005; Balemi and Negisho, 2012; Ingram et al., 2012). Unavailability of nitrogen and phosphorus in the soil changes RSA by modulating root cell elongation or cell division (Williamson et al., 2001; Smith and De Smet, 2012). Similar to the study of other abiotic stresses, we used germination pouch growth assays to investigate the effect of N and P deficiency on Setaria RSA. Seeds were germinated on pre-wet germination paper bags and supplemented with control MS media or MS media without N (MS-N, Caisson Labs) or without P (MS-P, Caisson Labs) 48 h post-imbibition. Seedlings were allowed to grow for 2 weeks and RSA as well as shoot phenotypes were examined. Both treatments resulted in a significant effect on the RSA of Setaria seedlings with ∼38 and 40% root growth inhibition in absence of N and P, respectively (**Figure 6D**). The young shoots of plants growing in the absence of N and P were pale green compared with the untreated seedlings.

### Adult Plant Growth and Phenotypes in Controlled Growth Chambers versus Greenhouse

**FIGURE 7:**
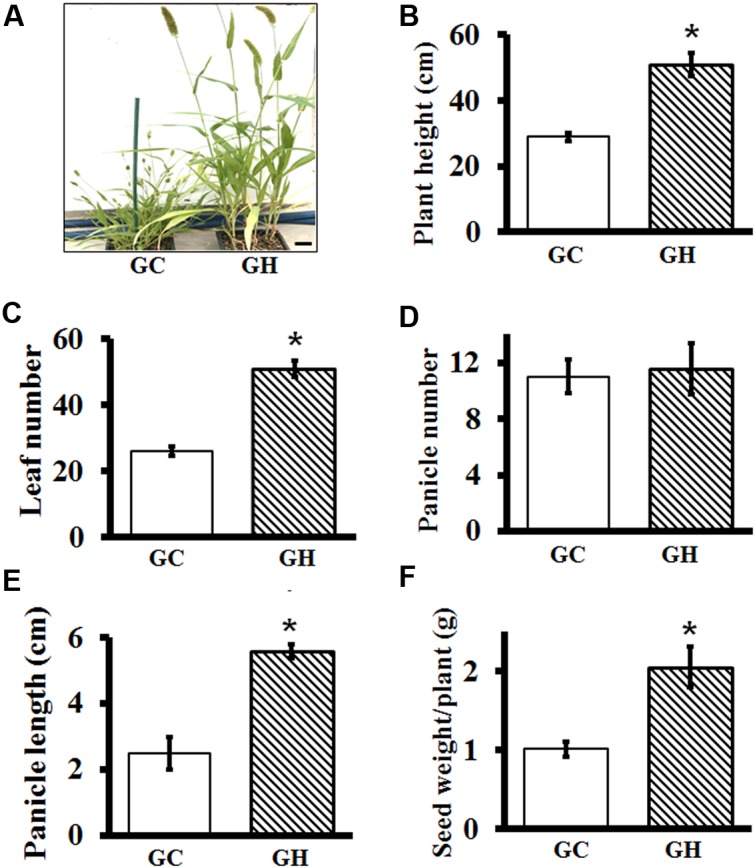
Comparative analysis of vegetative and reproductive growth parameters of Setaria grown in controlled environment growth chambers and in greenhouses. **(A)** Representative images of 7-week-old Setaria plants grown in growth chamber (GC, left) and greenhouse (GH, right). **(B)** Plant height at 7 weeks, **(C)** Leaf number at 4 weeks **(D)** panicle number at 7 weeks and **(E)** length of mature panicle at 7 weeks were measured. **(F)** Total seed weight was quantified from the plants grown under in growth chambers versus greenhouse. Three biological replicates of each treatment and four plants in each experiment were used for data analysis. Error bars represent standard errors (±SE). Asterisks represent *P*-values less than 0.05 as calculated using Student’s *t*-test. Scale bar is 5 cm.

For plants such as Setaria, where yield is determined by the number and size of panicles, it is important to precisely define the conditions that result in optimal growth. Similarly, to study the effect of different stress conditions on adult plants, control plants should grow in conditions that result in optimal growth and development. It is known that Setaria growth and development is extremely sensitive to photoperiod and temperature (Swanton et al., 1999; Doust et al., 2017). As expected, huge differences in overall plant architecture were observed between plants grown in controlled environment growth chambers versus controlled environment greenhouses. Plants grown in greenhouses were in general healthier and grew almost twice as large compared to the ones grown in growth chambers under similar conditions (except for the day length) (**Figure 7A**). To precisely compare the effect of different growth conditions, we grew a set of plants in growth chambers and greenhouses for the entire life cycle and documented key phenotypes. The overall conditions were similar in the greenhouse versus growth chamber and are listed in Supplementary Table S1.

Plants grown in greenhouses were taller (**Figure 7B**) and produced more leaves compared to the plants grown in the growth chambers, which were significantly smaller and had fewer leaves (**Figure 7C**). Panicle number per plant was not significantly different between the two growth conditions (**Figure 7D**); however, panicles of greenhouse grown plants were ∼3 times bigger (**Figure 7E**) and possessed more seeds. Consequently, yield, expressed as the seed weight per plant, was more than double in greenhouse-grown plants, compared to those grown in the controlled environment growth chambers (**Figure 7F**). This suggests that for the physiological experiments to be performed on adult Setaria plants, it may be important to grow them in conditions which result in their optimal growth.

### Effect of Different Stresses on Adult Plant Growth and Development

We evaluated the effects of two different stresses, low water potential and low nitrogen, on Setaria growth and development, singly and in combination. The water level of the low-water (L) plants was maintained at the 50% level of the control, well-watered (W) plants; and the low N plants did not receive any exogenous nitrogen whereas the nitrogen-sufficient plants had 15 mM (KNO_3_) added to the nutrient media. The stresses were applied from the time when the seedlings were 1-week-old and continued for the entire life cycle of the plant. The effects of single or combinatorial stresses were analyzed on overall plant architecture, plant height, leaf number, panicle number, and yield (expressed as total seed weight).

**FIGURE 8:**
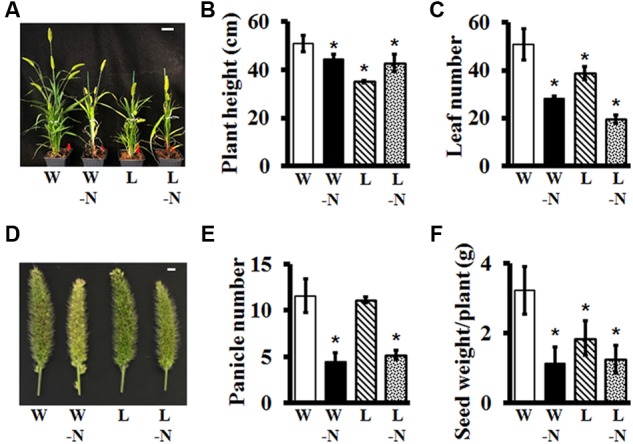
Effect of water and nitrogen deficit on vegetative and reproductive growth parameters of Setaria. **(A)** Representative image of 7 weeks old Setaria plants grown in the greenhouse under four different conditions: W = well-watered +15 mM nitrogen supplemented in the media, W-N = well-watered, no nitrogen supplement, L = low watered (50% compared to the well-watered control) +15 mM nitrogen, and L-N = low-water and no nitrogen supplement in the media. Different growth parameters such as **(B)** plant height and **(C)** leaf number were measured from seven and 4 weeks old Setaria plants, respectively. **(D)** Representative image of main panicle from 7 weeks old Setaria plants. **(E)** Panicles number per plant was counted from 7 weeks old plants. **(F)** Seed weight/plant was calculated after harvesting. Three biological replicates of each treatment and four plants in each experiment were used for data analysis. Error bars represent standard errors (±SE). Asterisks represent *P*-values less than 0.05 as calculated using Student’s *t*-test. Scale bars are 5 cm for **(A)** and 5 mm for **(D)**.

Setaria plants growing in soil in greenhouses grew fairly normally for the first 2 weeks of stress treatment, with no obvious differences when compared to control plants which were well-watered with nitrogen-containing nutrient media. However, the effects of different stresses became obvious as the plants grew and matured (**Figure 8A**). Low nitrogen led to pale coloration of the leaves, an effect that was more pronounced in the low nitrogen/low water combination stress. Overall plant height and biomass were significantly reduced in response to stresses (**Figure 8B**). Leaf number per plant was significantly lower in response to all stresses, but more pronounced in low nitrogen treatment with ∼50% reduction in response to low nitrogen and ∼60% reduction in response to low N in combination with low water (**Figure 8C**). Panicles exhibited unique characteristics in terms of color and size. Since Setaria is a relatively drought tolerant plant, it is not surprising that low water stress by itself did not have a huge effect on overall growth and development, panicle size or number of panicles per plant (**Figures 8D,E**); however, water-stressed plants did develop fewer seeds per panicle, which resulted in significantly reduced yield (**Figure 8F**). The effects of low nitrogen were more noticeable, both by itself and combined with water stress. These plants produced pale, thinner panicles, which were fewer in number and as a result had extremely poor yield (**Figures 8D–F**). These data suggest that even though Setaria is considered a wild, non-domesticated plant, it does exhibit consistent, obvious phenotypes in response to different stresses that can be used to characterize useful agronomic traits in various mutant populations, which will be valuable for their utilization in domesticated, food crops.

### Applications to Other *Setaria* Accessions

**FIGURE 9:**
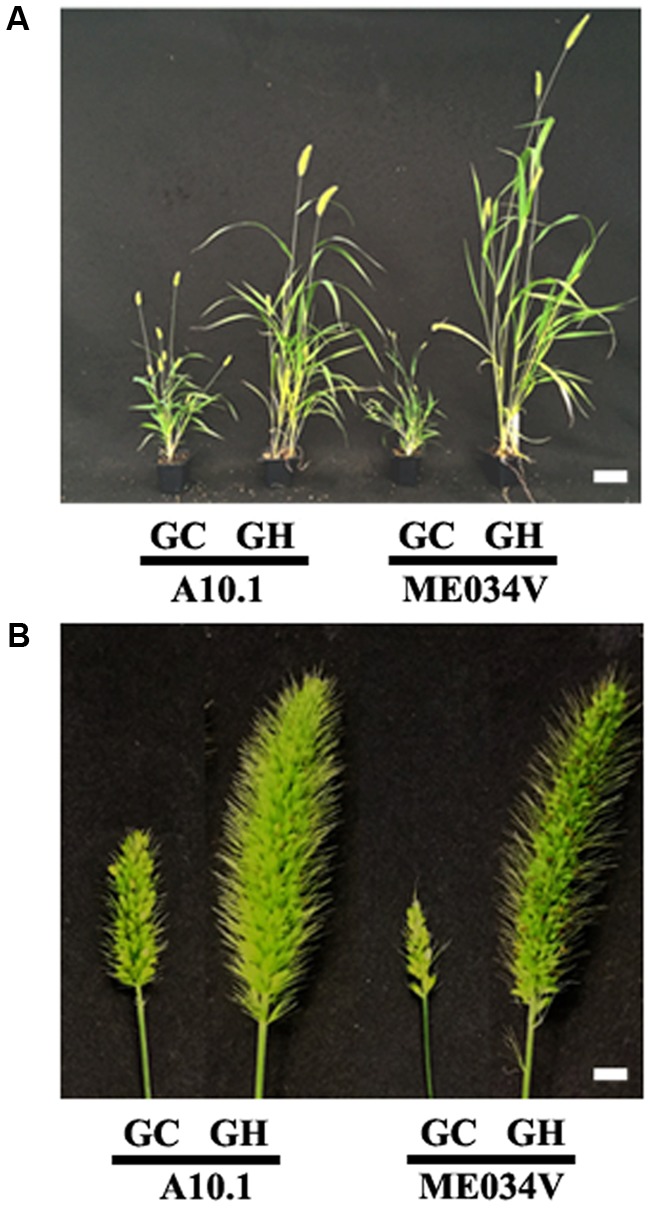
Comparison of Setaria accessions A10.1 and ME034V grown in controlled environment growth chambers versus in greenhouses. **(A)** Representative images of 5 weeks old Setaria plants A10.1 (left) and ME034V (right) grown in growth chamber (GC) and greenhouse (GH). **(B)** Representative images of A10.1 (left) and ME034V (right) panicles grown in growth chamber (GC) and greenhouse (GH). The plants were growth conditions under similar conditions in growth chamber and greenhouse under conditions listed in Supplementary Table S1. Scale bars are 5 cm for **(A)** and 5 mm for **(B)**.

Among more than 200 *S. viridis* accessions collected so far (Huang et al., 2014), A10.1 is of the greatest interest to the community because of the availability of different genetic tools, its sequenced genome (Bennetzen et al., 2012) and chemically induced mutant populations (Huang et al., 2017). Recently, increasing attention is also being paid to another accession ME034V, which is morphologically similar to A10.1 but shows significantly higher transformation efficiency (Zhu et al., 2017). Given the similarity of the two accessions, we believe that our findings with A10.1 can be applied to ME034V to a great extent. For example, we tested how ME034V grows in controlled growth chambers versus greenhouses. Similar to what we find in A10.1, ME034V grows much taller and healthier (**Figure 9A**) and produces much bigger panicles and more seeds (**Figure 9B**) in greenhouse conditions compared to in controlled growth chambers. This suggests that the methods presented in this paper can be used as a valuable reference for research using ME034V and other accessions in future.

## Conclusion

Optimization of multiple growth and development assays for Setaria, at three different stages of its life cycle, will be helpful for the research community to utilize it for more functional studies in the future. The standardization of different growth conditions, media, hormone concentrations, stresses and the description of resultant phenotypes will aid further targeted studies, and in combination with additional genetic and genomic tools being developed in multiple labs will greatly increase its use as a favorite model system for the study of grasses. Finally, a description of a set of optimized conditions will also help eliminate differences observed between different labs due to slightly altered conditions. These assays will serve as the first important steps to promote Setaria as a key model for a number of monocot species, where such resources are relatively limited.

## Author Contributions

SP, BA, and SRC designed research; BA, SRC, AE, AV, CZ, and LH performed research; BA, SRC, AE, AV, and SP analyzed data; BA, SRC, AV, CZ, and SP wrote the paper.

## Conflict of Interest Statement

The authors declare that the research was conducted in the absence of any commercial or financial relationships that could be construed as a potential conflict of interest.
