# Supplementary material for: Optimization of Phenotyping Assays for the Model Monocot Setaria viridis
Source: Front Plant Sci. 2017 Dec 22;8:2172. doi: 10.3389/fpls.2017.02172 (PMC5743732; doi:10.3389/fpls.2017.02172)
Supplement: Supplementary file 1 [file Table_1.DOCX]

**Table S1. Growth conditions for Setaria plants under controlled environment growth chambers versus greenhouses.**

| **Condition** | **Growth Chamber**  **(Conviron Model MTPC144)** | **Greenhouses**  **(1400 square feet)** |
| --- | --- | --- |
| **Light intensity-day** | **450 µmol/m^2^/sec** | **300-450 µmol/m^2^/sec** |
| **Temperature-day** | **31^o^C** | **28-30 ^o^C** |
| **Temperature-night** | **22^o^C** | **22-23 ^o^C** |
| **Day Length** | **12 h day/ 12 h night** | **14 h day/ 10 h night** |
| **Humidity** | **50-60%** | **30-50%** |
| **Pot size** | **10 cm** | **10 cm** |
